# Supplementary figures and images for: Impact of the DREAMS interventions on educational attainment among adolescent girls and young women: Causal analysis of a prospective cohort in urban Kenya
Source: PLoS One. 2021 Aug 12;16(8):e0255165. doi: 10.1371/journal.pone.0255165 (PMC8360512; doi:10.1371/journal.pone.0255165)

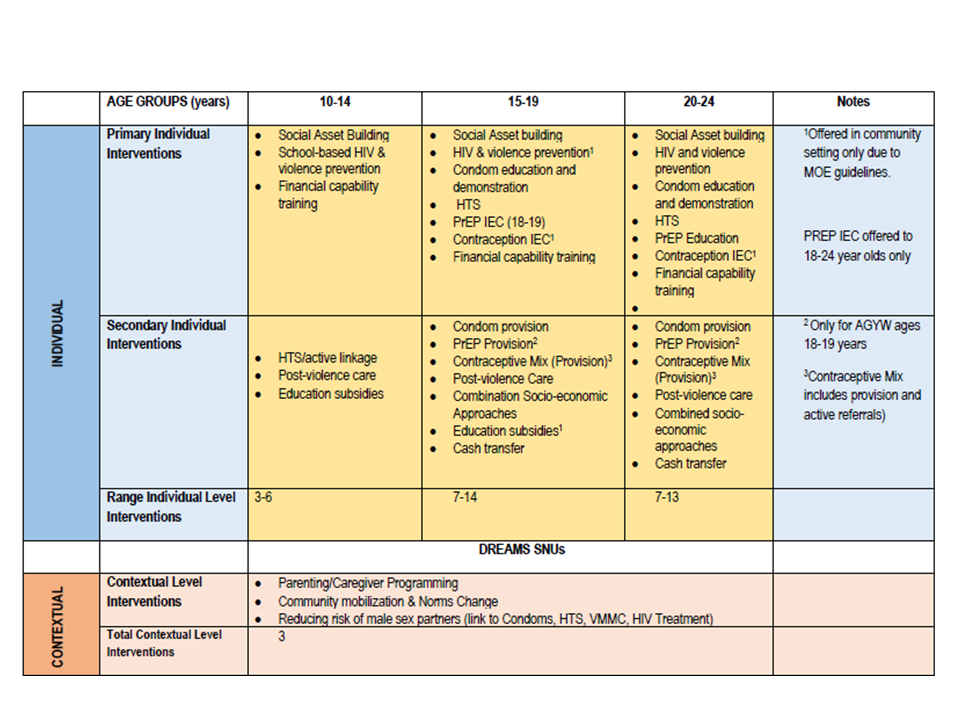

Supplement: S1 Fig — (TIF) [file pone.0255165.s001.tif]

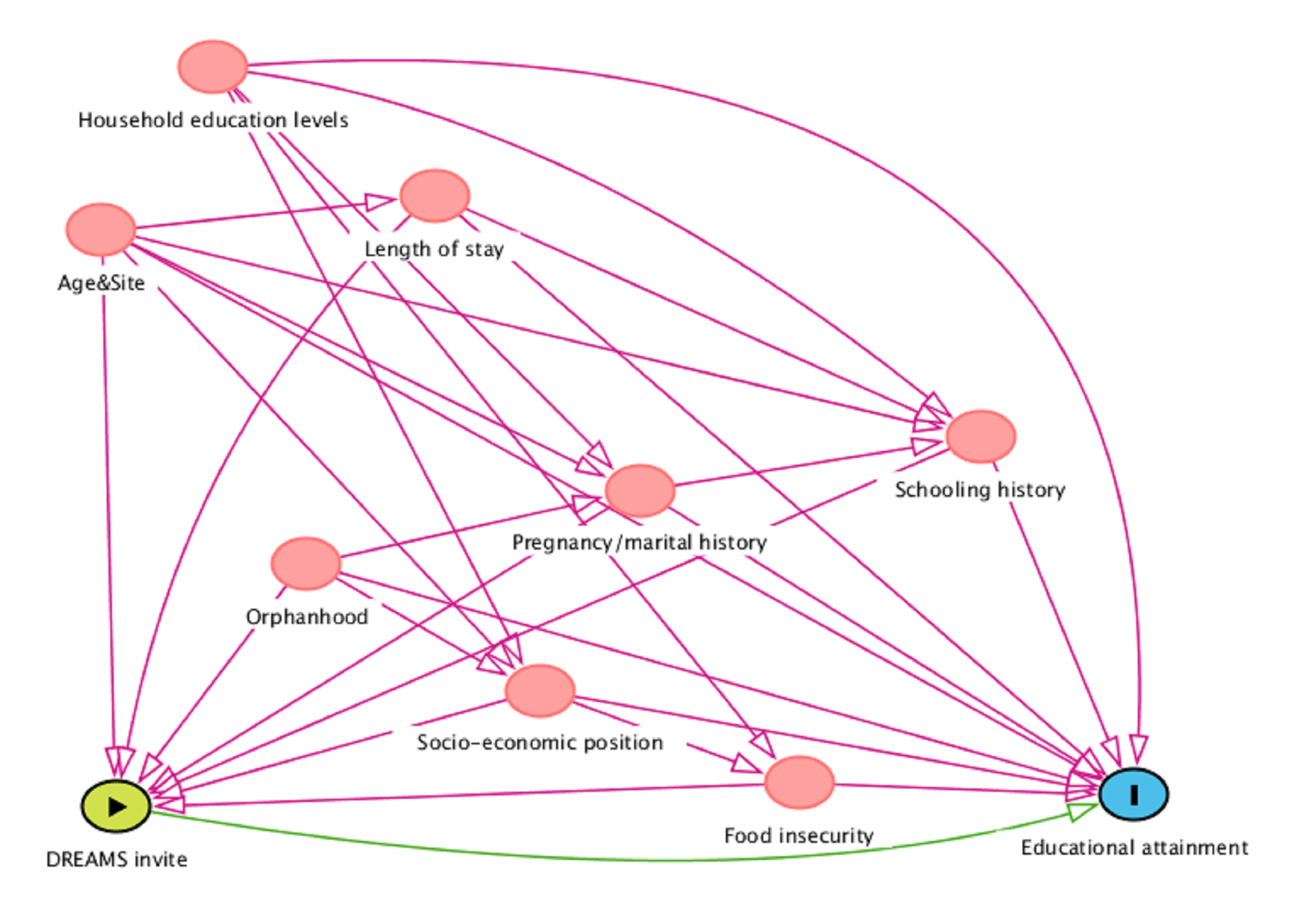

Supplement: S2 Fig — (TIF) [file pone.0255165.s002.tif]

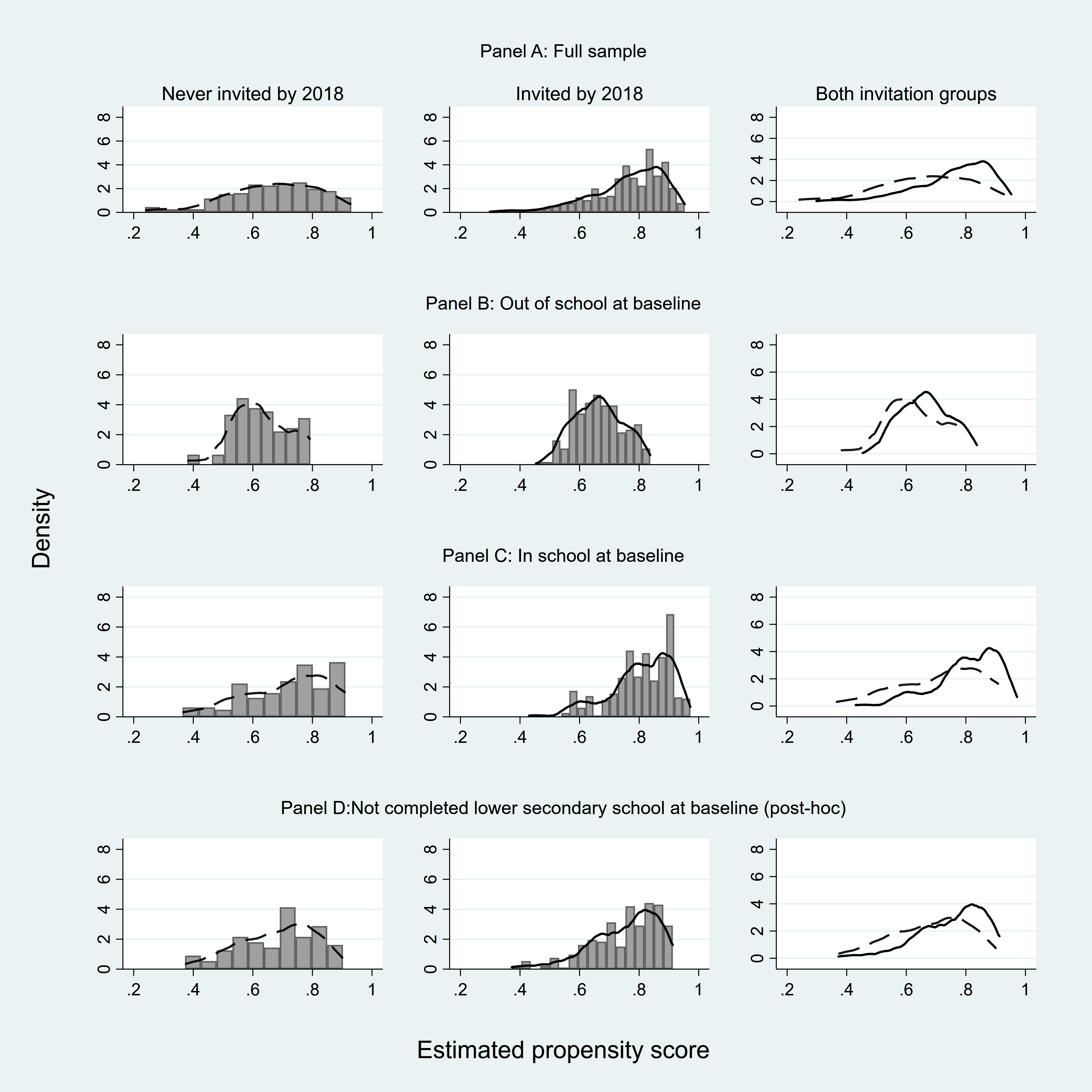

Supplement: S3 Fig — (TIF) [file pone.0255165.s003.tif]

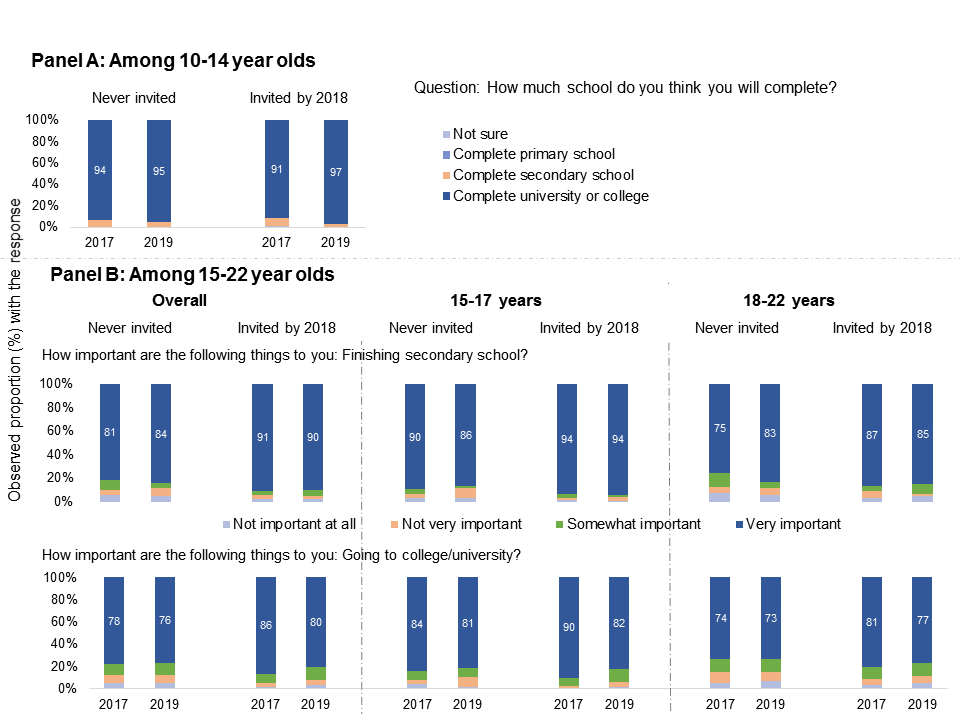

Supplement: S4 Fig — (TIF) [file pone.0255165.s004.tif]

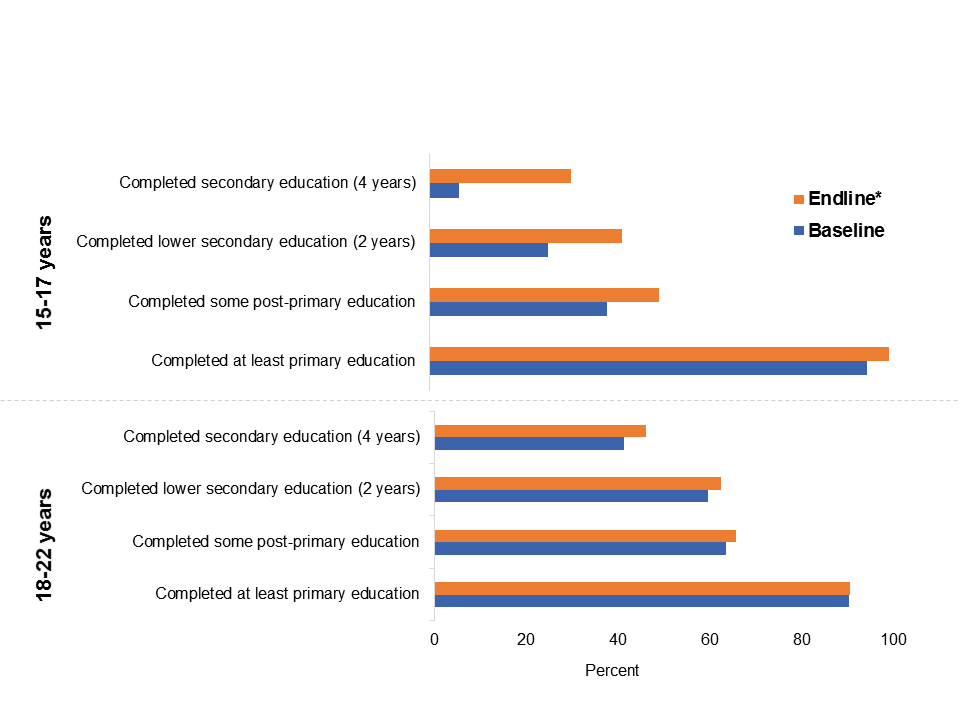

Supplement: S5 Fig — *Proportions at endline include those currently in school, as some participants re-enrolled during the follow-up. (TIF) [file pone.0255165.s005.tif]
