# Supplementary material for: Impact of the DREAMS interventions on educational attainment among adolescent girls and young women: Causal analysis of a prospective cohort in urban Kenya
Source: PLoS One. 2021 Aug 12;16(8):e0255165. doi: 10.1371/journal.pone.0255165 (PMC8360512; doi:10.1371/journal.pone.0255165)
Supplement: S1 Text — (DOCX) [file pone.0255165.s006.docx]

**DREAMS Impact Evaluation**

**An extract of the questions for nested cohort interviews & general population surveys: Round 3 (Nairobi)**

This tool is composed of a socio-behavioral module that will be administered to women and men aged 15 to 49 years in the NUHDSS as part of the General Population Component (GPC) AND the DREAMS Cohort Survey that will be administered to adolescent girls and young women aged 15 to 24 years.

**BACKGROUND INFORMATION**

| **A.1 FIELD WORKER'S CODE**   \|  \|  \|  \|  \|  \|  \|  \|  \| \| --- \| --- \| --- \| --- \| --- \| --- \| --- \| --- \|   **A.2 Site**   1. Korogocho 2. Viwandani   **DSS VILLAGE ____________________**  **A.3 DSS LOCATION ID**   \|  \|  \|  \|  \|  \|  \|  \|  \|  \|  \|  \| \| --- \| --- \| --- \| --- \| --- \| --- \| --- \| --- \| --- \| --- \| --- \|   **A.4 DSS HOUSEHOLD ID**   \|  \|  \|  \|  \|  \|  \|  \|  \|  \|  \|  \| \| --- \| --- \| --- \| --- \| --- \| --- \| --- \| --- \| --- \| --- \| --- \|   **A.5 HOUSEHOLD HEAD’S NAME _______________________________________**  **A.6 RESPONDENT’S ID**   \|  \|  \|  \|  \|  \|  \|  \|  \|  \|  \|  \|  \|  \|  \| \| --- \| --- \| --- \| --- \| --- \| --- \| --- \| --- \| --- \| --- \| --- \| --- \| --- \| --- \|     **A.7 RESPONDENT’S NAME** **_______________________________________**  **A.8 RESPONDENT’S GENDER**   1. Male 2. Female   **A.9 INTERVIEW VISITS**   \| **Interview visits** \| \| \| \| \| \| --- \| --- \| --- \| --- \| --- \| \|  \| **1** \| **2** \| **3** \| **Final visit** \| \| **Date**  **Interviewer name**  **Interview result** \|  \|  \|  \|  \| \| **Next visit: Date**  **Time** \|  \|  \|  \| **Total No. of visits** \|  \| \|  \|  \|  \|  \|  \|   **A.9a RESULT OF INTERVIEW**   1. Complete 2. Incomplete 3. Absent for extended period 4. Out-migrated 5. Refused 6. Structure located but whereabouts of respondent unknown 7. Parent/guardian refused 8. Dead 9. Incapacitated 10. Not age eligible 11. Other (specify) ______________________     **A.10 IS RESPONDENT SELECTED FOR THE COHORT STUDY**   1. Yes 2. No   **A.11 CONTACT INFORMATION** [FOR COHORT RESPONDENTS]  We would like to get your contacts, do you have a cellphone?   1. Yes 2. No   **A.12 RECORD CELLPHONE NUMBER**   \|  \|  \|  \|  \|  \|  \|  \|  \|  \|  \| \| --- \| --- \| --- \| --- \| --- \| --- \| --- \| --- \| --- \| --- \|   **99. Refused to share number**  **A.13 Do you have an alternate phone number that you can share in case we need to contact you in future**   1. Yes 2. No → A.16   **A.14 RECORD ALTERNATE PHONE NUMBER**   \|  \|  \|  \|  \|  \|  \|  \|  \|  \|  \| \| --- \| --- \| --- \| --- \| --- \| --- \| --- \| --- \| --- \| --- \|   **A.15 RECORD RELATIONSHIP OF PERSON WITH ALTERNATE NUMBER (i.e. whose number is this)**   1. Self 2. Spouse 3. Bio daughter/son 4. Step/adopt daughter/son 5. Bio mother/father 6. Step mother/father 7. Bio sister/brother 8. Step sister/brother 9. Maternal uncle/aunt 10. Paternal uncle/aunt 11. Maternal grandparent 12. Paternal grandparent 13. Cousin 14. Niece/nephew 15. Brother in-law 16. Sister in-law 17. Mother/father in-law 18. Spouse's other wife 19. Other relative (Specify) 20. Other non-relative (Specify)   **A.16 RESPONDENT’S RELATIONSHIP TO HOUSEHOLD HEAD**   1. Self 2. Spouse 3. Bio daughter/son 4. Step/adopt daughter/son 5. Bio mother/father 6. Step mother/father 7. Bio sister/brother 8. Step sister/brother 9. Maternal uncle/aunt 10. Paternal uncle/aunt 11. Maternal grandparent 12. Paternal grandparent 13. Cousin 14. Niece/nephew 15. Brother in-law 16. Sister in-law 17. Mother/father in-law 18. Spouse's other wife 19. Other relative (Specify) 20. Other non-relative (Specify)   **A.16a NUMBER OF PEOPLE CURRENTLY LIVING IN YOUR HOUSEHOLD ________**  **A.17 ENTER INTERVIEW DATE**:  **A.18 ENTER INTERVIEW START TIME [24 HOUR CLOCK]**: |
| --- | --- | --- | --- | --- | --- | --- | --- | --- | --- | --- | --- | --- | --- | --- | --- | --- | --- | --- | --- | --- | --- | --- | --- | --- | --- | --- | --- | --- | --- | --- | --- | --- | --- | --- | --- | --- | --- | --- | --- | --- | --- | --- | --- | --- | --- | --- | --- | --- | --- | --- | --- | --- | --- | --- | --- | --- | --- | --- | --- | --- | --- | --- | --- | --- | --- | --- | --- | --- | --- | --- | --- | --- | --- | --- | --- | --- | --- | --- | --- | --- | --- | --- | --- | --- | --- | --- | --- | --- | --- | --- |

**PART 1: SOCIO-DEMOGRAPHIC DATA**

| DSS & Cohorts | 1. **How old are you?**   RECORD AGE IN YEARS |
| --- | --- |
| DSS & Cohorts | 1. **When were you born?**   MONTH/YEAR *Ask respondent to give the best estimate if they do not know the year*  RECORD 98 IF DAY IS NOT KNOWN  RECORD 998 IF MONTH IS NOT KNOWN  RECORD 9898 IF YEAR IS NOT KNOWN |
| DSS & Cohorts | 1. **What is your religion?**   **INTERVIEWER’S NOTE:**   - **Roman Catholic** - **Protestants Churches- ACK, PCEA, Methodist, AIC, AIPCA, Lutheran** - **Pentecostal/ Charismatic- Full gospel, Charismatic Catholics, PAG, KAG and other with similar characteristics** - **Other Christians- Legio Maria, SDA, Jehovah Witness, Akorino etc.**  1. Catholic 2. Protestant 3. Pentecostal/Charismatic 4. Other Christian 5. Muslim 6. No religion 7. Other ____________________________ (specify) 8. Refused to answer |
| *DSS & Cohorts* | 1. **Which ethnic group do you belong to?** 2. Embu/Mbeere 3. Kalenjin 4. Kamba 5. Kikuyu 6. Kisii 7. Luhya 8. Luo 9. Maasai 10. Meru 11. Mijikenda/Digo/Rabai/Giriama/Duruma 12. Somali 13. Swahili 14. Taita 15. Taveta 16. Garre/Borana/Burji 17. Kuria 18. Teso 19. Other______ (specify) |

**Migration / Mobility**

| DSS & Cohorts | 1. **Where you were born?**      1. Korogocho 2. Viwandani 3. Non-DSA Nairobi slum 4. Nairobi non-slum 5. Other urban Kenya 6. Rural Kenya________ **(specify county- pull down menu)** 7. Outside Kenya 8. Don't know 9. Refused |
| --- | --- |
| DSS & Cohorts | 1. **How long have you lived in Viwandani/Korogocho?**   [IF LESS THAN ONE YEAR, RECORD ONLY THE NUMBER OF MONTHS]  2.Months  3.Years  0.Since Birth   1. Don’t know   ______ Y=Years;  ______M=Months; |
| DSS & Cohorts | 1. **In the last 3 months, have you stayed/lived in a place other than here (this site) for more than one month?**      1. Yes 2. No |
| Cohort | 1. **[IF 1.7 = YES] In the last 3 months, how many times have you stayed/lived in a place other than here for more than one month?**   _____________ Times   1. Don’t know 2. Refused to answer |
| Cohort | 1. **[IF 1.7 = YES] The last (most recent) time you lived/stayed in a place other than here for more than one month, why did you stay at this place?**      1. To visit my family (mother, father, sisters, brothers, aunts, uncles, grandparents) 2. To care for a sick relative (mother, father, sisters, brothers, aunts, uncles, grandparents) 3. For work 4. For a holiday or festival 5. To visit my children/family 6. To use medical services 7. To study/school-related reason 8. Other (Specify)__________ 9. Don’t know 10. Refused to answer |
| Cohorts | 1. **[IF 1.7 = YES] Where is the most recent place you stayed/lived for more than one month?**      1. Other slum in Nairobi 2. Other non-slum in Nairobi 3. Another city/urban area in Kenya 4. A rural area of Kenya________ **(specify county- pull down menu)** 5. Another country   98. Don't know  99. Refused to answer |
| Cohorts | 1. **[IF 1.7 = YES] The last (most recent) time you lived/stayed in a place other than here for more than one month, how long did you stay in this other place?**     1.Months  2.Years  98. Don't know  99. Refused to answer |
| Cohorts | 1. **In the last 3 months, have you lived/stayed in a household in this site (other than your usual household) for more than *one week*?** 2. Yes 3. No |
| Cohorts | 1. **[IF 1.12 = YES], in the last 3 months how many times have you stayed/lived in a household other than your usual household for more than one week?**     __________   1. Don’t know 2. Refused to answer |
| DSS & Cohorts | 1. **For how long do you intend to stay in Viwandani/Korogocho? ______**   [ **NOTE: Pick days if the respondent plans to move within the week, pick weeks if the respondent plans to move in one to three weeks, pick months if the respondent plans to leave in 1-11 months, pick years if the respondent plans to leave in one or more years.**  1. Days  2.Weeks  3.Months  4.Years  93. I do not plan to move  98. Don’t know  99. Refused to answer |
| DSS & Cohorts | 1. **[if respondent plans to move, Q1.14 is less than 1 year] Do you plan to move away from Viwandani/Korogocho permanently in the next 6 months?**      1. Yes   2. No |

**Marital status**

| DSS & Cohorts | 1. **Are you currently married or living together with a man/woman as if married?** 2. Yes, currently married 3. Yes, living with a man/woman 4. No, not in union   99. Refused to answer |
| --- | --- |
| DSS & Cohorts | 1. **[If 1.16 = No] Have you ever been married or lived together with a man/woman as if married?**      1. Yes, formerly married 2. Yes, lived with a man/woman 3. No 4. Refused to answer |
| DSS & Cohorts | 1. **[If 1.17= Yes, formerly married OR Yes, lived with a man/woman] What is your current marital status: are you widowed, divorced, or separated?**      1. Widowed 2. Divorced 3. Separated   99 Refused to answer |
| DSS & Cohorts | 1. **[if 1.16=Yes] Do you and your spouse/partner live in the same house?**      1. Yes 2. No |
| DSS & Cohorts | 1. **[IF 1.16=YES (Options 1 or 2) or 1.17=YES (Options 1 or 2)] How old were you when you first got married/started living with a partner as if married? [IF RESPONDENT DOESN’T KNOW] What is your best guess?**     __________ years old   1. Don’t know 2. Refused to answer |
| Cohorts | 1. **[IF 1.16=NO/REFUSED TO ANSWER or 1.17=NO/REFUSED TO ANSWER] Are you currently in a relationship?** 2. Yes 3. No   99. Refused to answer |

**Education**

| DSS & Cohorts | 1. **Have you ever attended school?**      1. Yes 2. No [SKIP TO 1.29] |
| --- | --- |
| DSS & Cohorts | 1. **Are you currently enrolled in school/college?** 2. Yes 3. No |
| DSS & Cohorts | 1. **[IF 1.24 = YES] What is the highest level of education you have completed?**      1. None 2. Primary Grade 1 3. Primary Grade 2 4. Primary Grade 3 5. Primary Grade 4 6. Primary Grade 5 7. Primary Grade 6 8. Primary Grade 7 9. Primary Grade 8 10. Secondary Form 1 11. Secondary Form 2 12. Secondary Form 3 13. Secondary Form 4 14. Vocational/trade school 15. College, certificate 16. University 17. Don’t know 18. Refused to answer |
| Cohorts | 1. **[IF 1.25 = YES] Within the last year of school/college, did you miss any term/semester? [if currently in school]**      1. Yes 2. No |
| Cohort | 1. **[IF 1.27 = YES] What is the main reason you missed the term/semester?**      1. Lack of school fees 2. Lack of school materials 3. Completed secondary/ completed college/university 4. Got pregnant 5. Got married 6. Sickness/illness/invalid/disabled 7. Domestic/household responsibilities 8. Not interested /bored with school 9. Not a good student 10. Got a job 11. Other (specify) 12. Don't know |

**Individual assets**

| DSS & Cohorts | 1. **Do you own/rent your own residence such as a structure or house?** 2. ***Y***es 3. No |
| --- | --- |
| DSS & Cohorts | 1. **[ IF 1.52= YES] In what month and year did you first own or rent your own house?**   *Ask respondent to give the best guess if they do not know the year*   1. Month ______ [Record 98=Don’t Know] 2. Year _______ [Record 98=Don’t Know] |
| Cohorts | 1. **I want to talk about the items that a person might own. Do you personally own or have these items?**      1. A blanket 2. A pair of shoes 3. School unifor 4. At least two sets of clothes (other than uniform) 5. A school bag 6. Some jewelry 7. Hair clips/ribbons 8. A mobile telephone 9. A clock or a wrist watch 10. A bicycle   Response Options   1. Yes 2. No   98. Don't know  99. Refused to answer |
| Cohorts | **Disability**   - - 1. **Do you have any of the following disabilities? [Allow selection of more than one]**  1. Sight (blind/severe visual impairment) 2. Hearing (deaf/profound difficulty in hearing) 3. Communication (speech impairment) 4. Physical (needs wheelchair/ crutches/stick) 5. Mental disability 6. Other specify   RESPONSE OPTIONS   1. Yes 2. No   97. Don't know  99.Refused to answer |
| Cohorts | **Orphanhood**  **1.54.2 Is your father alive?**   1. Yes 2. No [skip to 1.54.5]   98. Don't know [skip to 1.54.6]  99.Refused to answer |
| Cohorts | **1.54.3 [If 1.54.2=YES] Do you live with him?**   1. Yes 2. No   99.Refused to answer |
| Cohorts | **1.54.4 [If 1.54.2=YES] Is your father ill and perhaps needs caring for?**   1. Yes 2. No   99.Refused to answer |
| Cohorts | **1.54.4a [If 1.54.2a=YES] Are you involved in caring for your father?**   1. Yes 2. No   99.Refused to answer |
| Cohorts | **1.54.5 [If 1.54.2=NO] How old were you when your father died? [IF DON’T KNOW SKIP TO 1.54.5a]**  YEARS |
| Cohorts | **1.54.5a [If 1.54.5=DON’T KNOW] When did your father die?**  IF FULL DATE IS UNKNOWN ASK FOR MONTH/YEAR  *Ask respondent to give the best estimate if they do not know the year*  RECORD 98 IF DAY IS NOT KNOWN  RECORD 998 IF MONTH IS NOT KNOWN  RECORD 9898 IF YEAR IS NOT KNOWN |
| Cohorts | **1.54.6 Is your mother alive?**   1. Yes 2. No [SKIP to 1.54.9]   98. Don't know  99.Refused to answer |
| Cohorts | **1.54.7 [If 1.54.6=YES] Do you live with her?**   1. Yes 2. No   99.Refused to answer |
| Cohorts | **1.54.8 [If 1.54.7=YES] Is your mother ill and perhaps needs caring for?**   1. Yes 2. No   99.Refused to answer |
| Cohorts | **1.54.8a [If 1.54.8=YES] Are you involved in caring for your mother?**   1. Yes 2. No   99.Refused to answer |
| Cohorts | **1.54.9a [IF 1.54.6 =NO] How old were you when your mother died? [IF DON’T KNOW SKIP TO 1.54.9]**  YEARS |
| Cohorts | **1.54.9 [If 1.54.9a=DON’T KNOW] When did your mother die?**  IF FULL DATE IS UNKNOWN ASK FOR MONTH/YEAR  *Ask respondent to give the best estimate if they do not know the year*  RECORD 98 IF DAY IS NOT KNOWN  RECORD 998 IF MONTH IS NOT KNOWN  RECORD 9898 IF YEAR IS NOT KNOWN |

**Household food security**

| Cohorts | 1. **In the past 4 weeks, was there ever no food to eat of any kind in your house because of lack of resources to get food?** 2. Yes 3. No   98. Don't know  99. Refused to answer |
| --- | --- |
| Cohorts | 1. **In the past 4 weeks, did you or any household member go to sleep at night hungry because there was not enough food?** 2. Yes 3. No   98. Don't know  99. Refused to answer |
| Cohorts | 1. **In the past 4 weeks, did you or any household member go a whole day and night without eating anything at all because there was not enough food?** 2. Yes 3. No   98. Don't know  99. Refused to answer |
| Cohorts | 1. **In the last 4 weeks, were you or any household member not able to eat the kinds of foods you preferred because of a lack of resources?**      1. Yes 2. No   98. Don't know  99. Refused to answer |
| Cohorts | 1. **In the last 4 weeks, did you or any household member have to eat a limited variety of foods due to a lack of resources?**      1. Yes 2. No   98. Don't know  99. Refused to answer |
| Cohorts | 1. **Did you or any household member have to eat some foods that you really did not want to eat because of a lack of resources to obtain other types of food?**      1. Yes 2. No   98. Don't know  99. Refused to answer |
| Cohorts | 1. **In the last 12 months, did [you/any member of your household] ever get food aid from a church, NGO, the government or any other relief agency?** 2. Yes 3. No   98. Don't know  99. Refused to answer |

**Household structure**

| DSS & Cohorts | 1. **Main material of the floor**   [Observe and record. if not sure, ask respondent]  1. Natural floor Earth/sand  2. Natural floor- Dung  3. Rudimentary floor-Wood planks  4. Rudimentary floor-Palm/bamboo  5. Finished floor-Parquet or polished  6. Finished floor- Vinyl or asphalt strips  7. Finished floor Ceramic tiles  8. Finished floor Cement  9. Finished floor Carpet  10. Unable to observe  96. Other (specify) |
| --- | --- |
| DSS & Cohorts | 1. **Main material of the roof**   [Observe and record. if not sure, ask respondent]   1. Natural roofing -Grass / thatch / makuti 2. Natural roofing -Dung / mud 3. Rudimentary roofing- Corrugated iron (mabati) 4. Rudimentary roofing -Tin cans 5. Finished roofing- Asbestos sheet 6. Finished roofing Concrete 7. Finished roofing Tiles 8. Unable to observe 9. Rudimentary roofing- wood   96. Other (specify) |
| DSS & Cohorts | 1. **Main material of the wall**   [Observe and record. if not sure, ask respondent]  1. Natural walls-No walls  2. Natural walls-Cane/palm/trunks  3. Natural walls -Dirt/mud  4. Rudimentary walls- Bamboo with mud  5. Rudimentary walls -Stone with mud  6. Rudimentary walls- Uncovered adobe  7. Rudimentary walls Plywood  8. Rudimentary walls Cardboard  9. Rudimentary walls Reused wood  10. Rudimentary walls Corrugated iron (mabati)  11. Finished wall-Cement  12. Finished wall Stone with lime/cement  13. Finished wall Bricks  14. Finished wall Cement blocks  15. Finished wall Covered adobe  16. Finished wall Wood planks/shingles  77. Unable to observe  96. Other (specify) |
| DSS & Cohorts | 1. **What is the main source of drinking water for members of your household?**     1. Water Sellers/Vendors  2. Piped Water-Piped Into Dwelling  3. Piped Water Piped To Compound/Plot  4. Piped Water Public Tap/Standpipe  5. Well Water-Well On Residence/Plot  6. Well Water Public Well  7. Surface Water-River/Stream  8. Surface Water Pond/Lake  9. Surface Water Rainwater  10. Bottled Water  96. Other (specify) |
| DSS & Cohorts | 1. **What kind of toilet facility do your household members usually use?**     1. Flush Toilet-Own flush toilet  2. Flush Toilet Shared flush toilet  3. Pit toilet/latrine (Own traditional pit toilet  4. Pit toilet/latrine -Shared traditional pit toilet  5. Ventilated improved pit toilet-Own (VIP) latrine  6. Ventilated improved pit Shared (VIP) latrine  7. Flush trench toilet  8. Toilet without pit/working flush  9. Bucket/portable toilet  10. No facility/bush/field  11. Flying toilet  96. Other (specify) |
| DSS & Cohorts | 1. **What is the main method of garbage disposal used by your household?**      1. Garbage dump 2. In the river 3. On the road/along railway 4. In drainage/trench 5. In private pits 6. In public pits 7. Garbage disposal services 8. Vacant/abandoned house 9. Burning 10. No designated place/all over 11. Other____________________________(specify)   98. Don't know  99. Refused to answer |

**Demographic characteristics of household head**

| DSS & Cohorts | 1. **Highest education level of the head of your household. (only if the respondent is not the head of household)**   1. None 2. Primary Grade 1 3. Primary Grade 2 4. Primary Grade 3 5. Primary Grade 4 6. Primary Grade 5 7. Primary Grade 6 8. Primary Grade 7 9. Primary Grade 8 10. Secondary Form 1 11. Secondary Form 2 12. Secondary Form 3 13. Secondary Form 4 14. Vocational/trade school 15. College, certificate 16. University  17. Secondary Form 5  18. Secondary form 6  96. Other_____ (Specify)  98. Don’t know  99. Refused to answer |
| --- | --- |
| DSS & Cohorts | 1. **Is the head of your household male or female?**      1. Male 2. Female |

**Household assets**

| DSS & Cohorts | 1. **Do you have the following items in your household?**      1. Electricity 2. Refrigerator 3. Stove (electric, gas, or wood)/Jiko 4. Tap water in the house 5. Sofa set 6. Table 7. Bicycle 8. Motorcycle 9. Car/truck in working condition 10. Flash light (with working batteries) 11. Kerosene lamp with glass/lantern 12. Kerosene stove 13. Electric iron 14. Charcoal iron 15. Phone (cell or landline)) 16. Radio 17. Television   RESPONSE OPTIONS   1. Yes 2. No   98. Don't know  99. Refused to answer |
| --- | --- |

**Household poverty / well-being**

| DSS & Cohorts | 1. **In your opinion, how do you assess the economic situation of your household now?**      1. Very poor 2. Moderately poor 3. Not poor |
| --- | --- |

**Part 2. MEASURING EXPOSURE TO DREAMS**

| DSS & Cohorts | 1. **Have you heard of a program called ‘DREAMS’? DREAMS is an HIV prevention program targeting young girls and women that is being implemented by LVCT Health in Korogocho and Hope Worldwide in Viwandani**      1. Yes 2. No [skip to exposure matrix] | |
| --- | --- | --- |
| DSS & Cohorts | 1. **[IF 2.1=YES] From where / how did you hear about DREAMS?**      1. Mass media (TV, radio, newspaper) 2. Social media (web, Facebook, phone apps, email) 3. Word of mouth (a friend, relative, neighbor) 4. School 5. A health facility 6. A community-based or non-governmental organization (e.g., LVCT Health, Hope Worldwide) 7. A government agency 8. Community health workers 9. Other: _______________________________   MULTIPLE RESPONSES ALLOWED | |
| DSS & Cohorts | 1. **[IF 2.1=YES] Have ever you been invited to participate in any DREAMS activity or service?** 2. Yes 3. No   98. Don't know  99. Refused to answer  [If response is NO, DON’T KNOW OR REFUSED, skip to PART 3] | |
| DSS & Cohorts | **2.3a [IF 2.3=YES] Have you ever enrolled into/registered for DREAMS?**   1. Yes 2. No   98. Don't know |  |
| DSS & Cohorts | **2.3b [IF 2.3a=YES] When did you enrol/register**  **Month ________**  **Year ___________** |  |
| Cohorts | 1. **[IF 2.3=YES] What experience(s) have you had with DREAMS?**   OPEN ENDED RESPONSE |  |
| DSS & Cohorts | 1. **[IF RESPONDENT IS FEMALE AGED 10-24 YEARS AND THOSE WHO SAID YES TO 2.3] Do you have a DREAMS identifier/Unique Identification Number (UIN)/Ukona Namba ya mradi? It could be a reference number, a card or badge, or code.**      1. Yes 2. No   98. Don't know  99. Refused to answer |  |
| DSS & Cohorts (Only AGYW) | **2.5a [For those who said Yes to 2.5] Are you willing to share your DREAMS ID [number or identifier] with us? We would like this so that we can look at your use of DREAMS interventions. All of the information that you give us will be kept confidential.**   1. Yes, Record Number _______________________ 2. Yes, but I don’t know the number 3. No   98. Don't know  99. Refused to answer |  |
| DSS & Cohorts (Only AGYW) | **2.5b [For those who said Yes to 2.5a] Field staff to record the source of the girl’s DREAMS ID number?**   1. Memory 2. Presented on a document/card/badge   96. Others ______(specify)  **[Field staff can use these to complete 2.3b** |  |

**DREAMS Exposure Matrix**

| **Age/Sex of participants to be asked in the DSS** | **Type of service or program in the DREAMS core package** | **a. Are you aware of the following service / program?**  1. Yes  2. No  IF NO SKIP TO NEXT SERVICE | **b. Have you ever used (participated in) the service / intervention?**  1. Yes  2. No  98. Don't know  99. Refused to answer  IF NO SKIP TO “g” | **If ever used or participated in the intervention:** | | | | | | **g. If never used: why not?**  ***?***  1. Didn’t need it  2. Haven’t heard of it  3. Not available to me  96. Other(Specify)  98. Don't know  99. Refused to answer | | |
| --- | --- | --- | --- | --- | --- | --- | --- | --- | --- | --- | --- | --- |
|  |  |  |  | **c. Did you use/ participate in the service in the past 12 months?**    1. Yes  2. No  98. Don't know  99. Refused to answer  IF NO GO TO NEXT SERVICE | **d. * How many sessions/times did you participate in?** | | **e. When you used the service in the last 12 months, was it a ‘DREAMS’ program?**  1. Yes  2. No  98. Don't know  99. Refused to answer | | **f.** **From which organization was it provided?** |  |  |  |
| AGYW | 1. Safe Spaces program for girls/young women* |  |  |  |  | |  | |  |  | | |
| ALL | 1. HIV testing and counselling services at a health facility; mobile clinic; home; community;NGO/LVCT self-tested or safe space, school |  |  |  |  | |  | |  |  | | |
| ALL | 2.7a Partner testing (HIV testing with your sexual partner) |  |  |  |  | |  | |  |  | | |
| ALL | 1. Condom provision at health facilities, home or in community. |  |  |  |  | |  | |  |  | | |
| ALL | 2.8a Condom promotion, education and demonstration |  |  |  |  | |  | |  |  | | |
| Males in the DSS | 1. Voluntary medical male circumcision |  |  |  |  | |  | |  |  | | |
| ALL | 1. Counselling on and provision of contraception / family planning method |  |  |  |  | |  | |  |  | | |
| ALL | 1. Post-violence care counseling and services (e.g., HIV and STI testing, or linkage with the legal system) |  |  |  |  | |  | |  |  | | |
| ALL | 2.11a HIV and STI testing services after an experience of violence (including sexual, physical or emotional violence) |  |  |  |  | |  | |  |  | | |
| ALL | 2.11b Any other post-violence care services (e.g., legal or police) |  |  |  |  | |  | |  |  | | |
| ALL | 2.11c Post-exposure prophylaxis (PEP) (Drugs that can be taken to prevent HIV infection *after* possible exposure to the virus, including post-violence) |  |  |  |  | |  | |  |  | | |
|  | 2.11d Pre-exposure prophylaxis (PrEP) (Drugs that can be taken to prevent HIV infection *before* possible exposure to the virus) |  |  |  |  | |  | |  |  | | |
| ALL (in school) | 1. School-based HIV education/programs, such as life skills, Health Choices; Shuga; Tupange; My Health My Choice; violence prevention & gender norms*(No means No) |  |  |  |  | |  | |  |  | | |
| ALL | 1. Cash transfer to family / household or AGYW |  |  |  |  |  | |  | | |  |  |
| AGYW | 1. Educational subsidies (e.g., support for school fees, uniforms, books, or stationery) to help girls stay in school |  |  |  |  |  | |  | | |  |  |
| AGYW | 1. Microfinance program |  |  |  |  |  | |  | | |  |  |
| AGYW | 1. Financial literacy training for girls/young women* |  |  |  |  |  | |  | | |  |  |
| AGYW | 1. Savings group / table banking* |  |  |  |  |  | |  | | |  |  |
| AGYW | 1. Vocational/ business skills training* |  |  |  |  |  | |  | | |  |  |
| All | 1. Violence prevention-related training or education in the community such as SASA, gender norms training* |  |  |  |  |  | |  | | |  |  |
| All  Need a filter question for parenting/caregiving | 1. Parent/care-giver ‘positive caregiving’ program/ Families Matter!* |  |  |  |  |  | |  | | |  |  |
| All | 1. Have you heard of /participated in any other program designed to encourage healthy, HIV-Free living? |  |  |  |  |  | |  | | |  |  |
| AGYW | 1. Ajiri dada (by AMURT) |  |  |  |  |  | |  | | |  |  |
|  | 1. Tackle Africa for adolescent men in Korogocho (by MYSA) |  |  |  |  |  | |  | | |  |  |
|  | 1. Ushahidi |  |  |  |  |  | |  | | |  |  |
| AGYW | 1. Wezesha Dada |  |  |  |  |  | |  | | |  |  |

**Part 3. QUESTIONS TO MEASURE MEDIATING VARIABLES**

**Aspirations (Future orientation, Expectations)**

| Cohorts | 1. **The next questions are about some goals that you may have. How important are the following things to you (READ STATEMENT). Would you say (READ THE CHOICES): Not important at all, not very important, somewhat important, very important**      1. Finishing secondary school. 2. Going to college/university. 3. Owning your own home. 4. Helping to take care of your parents or family when you are older. 5. Being admired and respected by your friends. 6. Having a good job/stable income 7. Having children 8. Getting married or finding a partner   RESPONSE OPTIONS   1. Not important at all 2. Not very important 3. Somewhat important 4. Very important |
| --- | --- |
| Cohorts | 1. **What are the chances that (READ EACH STATEMENT). Would you say the chances are high (or already achieved), about 50/50, or low?** 2. You will finish primary school 3. You will join secondary school? 4. You will finish secondary school? 5. You will go to university? 6. You will have a job that pays well? 7. You will be able to own your own home? 8. You will have a job that you enjoy doing? 9. You will have a happy family life? 10. You will stay in good health most of the time? 11. You will live in safe neighbourhood 12. You will be respected in your community   RESPONSE OPTIONS   1. High (or already achieved) 2. About 50/50 3. Low |

**Part 4. QUESTIONS ON SEXUAL BEHAVIOUR OUTCOMES**

| GPC & Cohorts | 1. **Have you ever had sex, even if it was forced?**      1. Yes 2. No 3. Refused to answer   If response is NO or REFUSED, skip to 4.75 |
| --- | --- |
| GPC & Cohorts | 1. **If you have ever had sex (even if it was not your choice), how old were you the first time that it happened? [IF RESPONDENT DOESN’T KNOW] What is your best guess?**   RECORD AGE IN YEARS  RECORD 98 IF AGE IS NOT KNOWN |
| DSS & Cohorts | 1. **Have you had sex in the past 12 months?** 2. Yes 3. No   99. Refused to answer |
| DSS & Cohorts | 1. **[IF 4.15=YES] In the past 12 months, how many different people have you had sex with? [IF RESPONDENT DOESN’T KNOW] What is your best guess?**   RECORD NUMBER OF PEOPLE  RECORD 998 IF RESPONDENT DOES NOT KNOW THE NUMBER  RECORD 999 IF REFUSED TO ANSWER |

**Pregnancy**

| DSS & Cohorts | **4.86 [If 4.1=YES] Have you ever been pregnant/ Have you ever made someone pregnant?**   1. Yes 2. No   99. Refused to answer |  |
| --- | --- | --- |
| DSS & Cohorts | **4.87 [IF 4.86=YES] How old were you the first time you got pregnant/made someone pregnant?**  RECORD AGE  RECORD 98 IF AGE IS NOT KNOWN  RECORD 99 IF RESPONDENT REFUSES TO ANSWER |  |
| DSS & Cohorts | **4.88 [IF 4.86=YES & RESPONDENT IS FEMALE] How many times have you been pregnant? (including pregnancies that did not go to full-term / result in a birth)**  RECORD NUMBER OF TIME  RECORD 98 IF DON’T KNOW |  |
| DSS & Cohorts | **4.89 [IF 4.86=YES AND RESPONDENT IS FEMALE] How many times have you given birth? (Include both live births and stillbirths)**  Multiples, such as twins/triplets, count as 1 birth)  RECORD NUMBER OF TIMES  RECORD 98 IF DON’T KNOW |  |
| DSS & Cohorts | **4.89a [IF 4.86=YES AND RESPONDENT IS FEMALE] Have you given birth in the last 12 months?**   1. Yes 2. No   99. Refused to answer |  |
| DSS & Cohorts | **4.90 [IF 4.86=YES] How many children do you have?**  RECORD NUMBER OF CHILDREN  RECORD 98 IF DON’T KNOW [ONLY FOR MALES] | |
| DSS & Cohorts | **4.91 [IF 4.86=YES AND RESPONDENT IF FEMALE] Are you currently pregnant?**   1. Yes, I am pregnant 2. Not pregnant   98. Don’t know | |

**Distress screening to be asked at the end of survey (link to respondent ID)**

*“We are now finished with the questions. Thank you so much for talking to me, you did a really great job!”*

**Please call over the interviewer to finalize your survey. [Enter finalization code “2015”]**

**close**  “**2015**” Code to close out survey

**dstrss1 D1. “I know the some of the questions that I asked may have been sensitive or uncomfortable for you to talk about. Can you tell me how are you feeling right now?”**

**[Interviewer should fill out the below options based on what the respondent tells them (e.g. ok, good, worried, upset) or their impression of how the respondent is feeling]**

1. Good (happy, not at all upset)
2. Ok (not happy or upset)
3. Somewhat worried/upset
4. Very worried/upset
5. Reported abuse

**dstrss1a [If dstrss1=2 or 3, ask:] If you are comfortable telling me, please tell me what has upset, worried or made you uncomfortable? (string)**

_______________________________________________________________

**[If dstrss1=3 or 4, ask:] D2. If the respondent reports abuse or that they are very worried/upset:**

**“Based on your saying to me [or showing] that our interview may have upset you, I would like to share this with my supervisor [field coordinator] so that we can let you know where to find help that might be useful. [For minors] If ok with you, we will also talk to your mother (or father) so that they can help you” [If the adolescent does not want to share this with his/her parents/guardians, help them identify another adult they could talk with].**

**dstrss2** Action taken: __________________________________________________________

**D3. If the respondent does not report abuse, and is not very worried/upset:**

**“We have talked about many things today that you might have more questions about [give examples e.g. romantic relationships, sex, bullying or violence]. I want to give you this card with numbers and locations for organizations [say the local names] that work with young people your age. You might have heard of some, and some might be new to you. If you have questions or want to talk to someone, you can call them and they will try to help you.”**

**Interviewer assessment**

**Interviewer, please complete the questions below based on your own observation and assessment of the entire interview process, and the respondent.**

**dstrss4 D4. How did you find the respondent’s cooperation?**

1. Very good
2. Moderate (ok)
3. Bad
4. Very bad

**dstrss4a [If dstrss4=4] Please explain why very bad _________ (string)**

**dstrss5 D5. How accurate/true did you find the respondent’s answers?**

1. Very accurate/true
2. Somewhat accurate/true
3. Not very accurate/true
4. Highly inaccurate (the responses should not be trusted)

**dstrss5a [If dstrss5=4] Please explain why can’t be trusted _________ (string)**

**dstrss6 D6. How did you find the respondent’s understanding of the questions discussed?**

1. Very good (understood perfectly)
2. Moderate (understood ok)
3. Bad (did not understand many of the questions)
4. Very bad (did not understand at all)

**dstrss6a [If dstrss6=4] Please explain about their not understanding_________ (string)**

**dstrss7 D7. How did you find the respondent’s concentration and attentiveness during the interview?**

1. Very good (highly concentrated/attentive)
2. Moderate/ok (somewhat concentrated/attentive)
3. Bad (could not concentrate for many parts of the interview)
4. Very bad (could not concentrate at all)

**dstrss7a [If dstrss7=4] Please explain why very bad at concentration_________ (string)**

**dstrss8 D8. About how many breaks did you take during the full interview?**

______ Number of breaks

**dstrss9 D9. Other comments about the interview** __________________________________________________________________________________________________________________________________________

**This concludes our interview. Thank you very much for your time.**
