# Supplementary material for: Impact of the DREAMS interventions on educational attainment among adolescent girls and young women: Causal analysis of a prospective cohort in urban Kenya
Source: PLoS One. 2021 Aug 12;16(8):e0255165. doi: 10.1371/journal.pone.0255165 (PMC8360512; doi:10.1371/journal.pone.0255165)

**Causal interpretation and sensitivity analyses**

To ensure the propensity score analyses produce valid causal estimates for the educational attainment (as defined in the methods – see *attainment 3*), five key assumptions are necessary:

1. no interference – In the context of educational attainment, this assumption means that one individual’s educational attainment should not be affected by another individual’s invitation (or non-invitation) to DREAMS. Interference may arise from within households in low resource settings. For instance, if a household has two or more AGYW, and one of them receives educational subsidies through DREAMS, this may free up resources for the other siblings for more schooling. Such instances are likely to be very limited in the study context, however.
2. positivity – all individuals must have a non-zero chance of being exposed or unexposed to DREAMS. While DREAMS was targeted, the implementation of targeting was carried out in such a way that there was a non-zero probability that an individual in the cohort could receive or not receive DREAMS.
3. correct specification of the propensity score model – we considered all variables which were related to the exposure and/or the outcome, and all variables in the propensity score model were included as categorical variables. In all models (propensity score regression adjustment, propensity score stratification and inverse probability weighting with the propensity score (IPW)), we used the same propensity score prediction model. Covariate balance statistics of the measured confounders indicate the balancing property was achieved after weighting and stratification, suggesting the specification was appropriate.
4. conditional exchangeability – theoretically and conditional on the covariates in the model, the exposed and unexposed groups should be interchangeable i.e., replace one group with the other without affecting the outcome. Unmeasured confounding cannot be ruled out in our analysis, but the potential bias was minimised by carefully including at least one covariate from the constructs identified in the causal graphs.
5. consistency – this assumption requires that exposure to DREAMS is clearly defined, such that any variations in receiving DREAMS would not result in a different outcome. These analyses used a clear definition of exposure to DREAMS: invited yes or no. DREAMS implementation was based on a coherent core-package of interventions, and context specific adaptations were allowed. The impact of this heterogeneity on educational attainment is likely to be minimal given the methods of intervention delivery in Nairobi – the delivery of DREAMS was fairly consistent in each setting, as implementation was coordinated by one implementing partner over the same time frame, and prioritization strategies to recruit the most vulnerable girls and young women evolved in a similar way across the two settings.

Covariate balancing checks were done only for inverse probability weighting with the propensity score. Standardised mean differences using ‘covbal’ command in Stata were used to assess the balancing property. We computed the standardised mean differences before and after accounting for the inverse probability weights. Results indicate good balancing after weighting (Figure 1).

**Figure 1: Covariate balance mean differences**


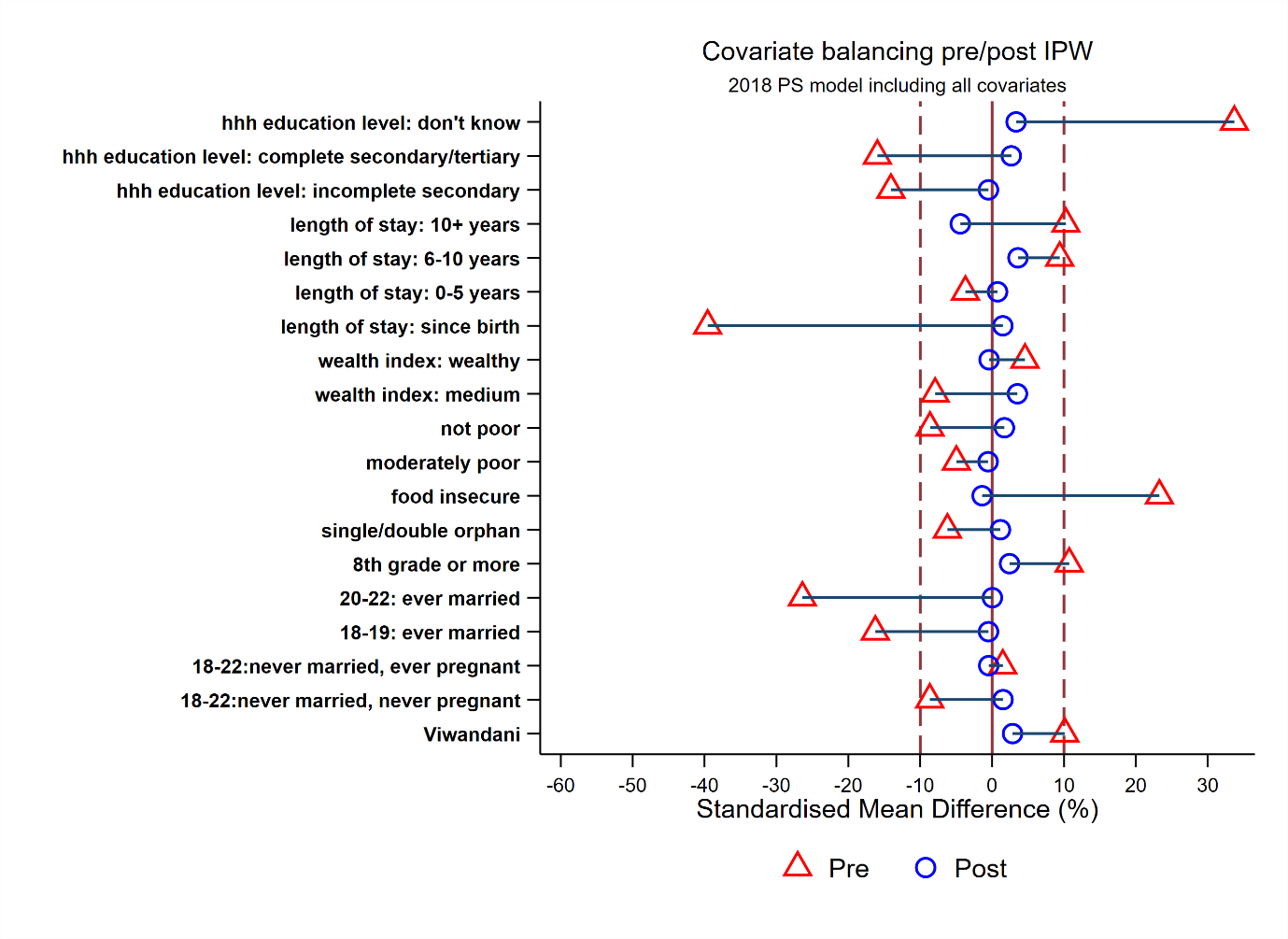

Supplement: S2 Text — (DOCX) [file pone.0255165.s007.docx]
